# Alveolar soft part sarcoma: clinicopathological findings in a series of 11 cases

**DOI:** 10.1186/1477-7819-6-71

**Published:** 2008-07-01

**Authors:** Adrien Daigeler, Cornelius Kuhnen, Joerg Hauser, Ole Goertz, Daniel Tilkorn, Lars Steinstraesser, Hans-Ulrich Steinau, Marcus Lehnhardt

**Affiliations:** 1 Department of Plastic Surgery, Burn Center, Hand surgery, Sarcoma Reference Center, BG-University Hospital Bergmannsheil, Ruhr University Bochum, Buerkle-de-la-Camp-Platz 1, 44789 Bochum, Germany; 2 Institute for Pathology, BG-University Hospital Bergmannsheil, Ruhr-University Bochum, Bürkle-de-la-Camp-Platz 1, 44789 Bochum, Germany

## Abstract

**Background:**

Alveolar sarcoma of the soft parts (ASPS) represents a very rare entity of soft tissue sarcoma with special features such as young peak age incidence and frequent metastasis to the brain. The aim of this study was a clinicopathological analysis with special reference to treatment and outcome.

**Methods:**

From the database of the BG-University Hospital Bergmannsheil, 1597 soft tissue sarcoma (STS) cases were reviewed and 11 consecutive patients with ASPS were isolated. Data was acquired from patients' charts and contact to patients, their relatives or general practitioners, with special reference to treatment and clinical course. The average follow up time from the time of the definite operation for the primary tumor was 6.5 years. Kaplan-Meier method was used to calculate survival.

**Results:**

Patients with localized disease who received complete resection and adjuvant radiation and who did not develop recurrence or metastatic disease within 2 years after surgery had a positive outcome. The size of the tumor, its localization, and the time of untreated growth before treatment did not influence the long-term results. All patients who developed recurrent disease also suffered from distant metastasis, reflecting the aggressive biology of the tumor. All patients with distant metastasis had the lungs and the brain affected.

**Conclusion:**

Due to the limited number of patients with ASPS, prospective studies would have to span decades to gather a significant collective of patients; therefore, it is not possible to comment meaningfully on a possible benefit of neoadjuvant or adjuvant therapy.

We recommend wide surgical excision and, in the absence of data telling otherwise, adjuvant radiation. In cases with recurrent disease or metastasis, the prognosis is bad and further treatment will be restricted to palliation in most cases.

## Background

Alveolar soft part sarcoma (ASPS) is a very rare type soft tissue sarcoma (STS), with several unusual features, such as a very young peak age incidence and frequent metastatic spread to the brain [1]. Accounting for less than 1% of STS, it presents at almost every part of the body with a predominance of the trunk and the proximal extremities [2-5] and usually affects patients younger than 40 years [5]. The name "alveolar" was derived from its pseudo-alveolar appearance with clustered polygonal cells lacking central cohesion [4]. Recent cytogenetic studies revealed chromosome rearrangements at t(X;17)(p11;q25) resulting in the ASPL-TFE3 fusion gene, but the origin of ASPS still remains unclear, in fact, it seems that a normal cellular counterpart for this sarcoma does not exist [6-9]. Due to its rarity, its unusual clinical course and an indolent progression of disease diagnosis and treatment has been proven to be a challenge for the pathologist and the surgeon as well. We reviewed our single center experience with ASPS over a period of 16 years with special reference to the clinical course and outcome and assessed our findings against the background of the existing literature.

## Methods

From 1991 to 2007, 11 out of 1597 patients that were treated at our institution for STS were diagnosed with alveolar sarcoma of the soft parts (ASPS). Data for this case series were acquired retrospectively from the sarcoma database of BG-University Hospital Bergmannsheil. Additional information regarding the clinical course and outcome was collected from the patients' charts, and phone calls to the patients, their relatives and their general practitioners. Follow-up data were available for all patients and consisted of clinical examination, chest X-ray or computed tomography, abdominal ultrasound and CT or MRI of the tumor site and the brain in three cases. Local recurrence was defined as tumor occurrence after treatment at a site of previous operation. Metastasis was diagnosed when the tumor occurred at any other site. Summary statistics were obtained using the Kaplan-Meier method for calculating survival. Because of the low number of patients we refrained from further statistical analysis.

Four patients were female, seven were male, and the average age at time of diagnosis was 32 years (range: 19–49). The follow-up time from the time of the definite operation for the primary tumor was 78 months/6.5 years (5–156 months).

### Histopathological examination

In all cases the diagnosis of ASPS was confirmed by a review of the pathology slides by experienced soft tissue pathologists of our institution. In two cases (patient 5 and 9), tissue specimens were sent in for second opinion to another experienced soft tissue pathologist. In both cases, the primary diagnosis (ASPS) of our institution was confirmed.

## Results

With 11 cases of alveolar sarcoma of the soft parts out of 1597 patients with soft tissue sarcoma, ASPS accounted for 0.7% of STS in our data base. Ten patients could remember the period of time the tumor was growing before definite diagnoses was made. This time ranged from one month (patient 11) to 20 years (patient 5). A correlation between the duration of untreated tumor growth and outcome could not be detected.

The site most often affected by the tumor was the thigh (n = 4) followed by the lower leg (n = 2) and the thoracic wall (n = 2), the upper arm, the forearm, and the foot in one case each. All tumors were located intramuscular or subfascial with an average of 6.8 cm in largest diameter (range: 2.9–13.5 cm). All patients with an unfavorable outcome had tumors below the average size (table 1).

**Table 1:** Summarized tumor data.

Patient	Localisation	Size in cm	TNM classification	Comments	Status
1	left thigh, intramuscular	10 × 8 × 4	ypT2 N0 M0	70% vital tumor in resection specimen: none responder	alive, NED
2	right thigh, intramuscular	5.5 × 4	pT2 N0 M0	2 tumor free lymph nodes in primary specimen, lymphangiosarcomatis	DOD
3	right upper arm, intramuscular	5.6 × 4.7 × 3.3	pT2 N0 M0	-	DOD
4	left thigh, intramuscular	8 × 6,5 × 5	pT2 N0 M0	3 tumor free lymph nodes in primary specimen, second expert opinion by Dr. Mentzel (Friedrichshafen, Germany) and Prof. Fletcher (Boston, USA)	alive, NED
5	right lower leg, intramuscular	13,5 × 9,5 × 8,3	ypT2b N0 M0	-	alive, NED
6	right dorsum, intramuscular	3,5 × 2,9 × 1.9	pT1 N0 M0	-	alive, NED
7	left thigh, intramuscular	10.5 × 7.3 × 5.3	pT2 N0 M0	-	alive, NED
8	left heel, subfascial	4 × 3 × 2.5	pT1 N0 M0	-	DOD
9	right lower leg, intramuscular	3.5 × 2.7 × 1.7	pT1b N0 M0	Inguinal dissection because of suspicious lymph nodes, ruling out lymph node metastasis, second expert opinion by Prof. Katenkamp, Jena (Germany)	alive, NED
10	left thorax, subscapular, intramuscular	7.8 × 2.5 × 1.3	pT2b N0 M0	-	alive, NED
11	right forearm, intramuscular	2.9 × 2.4 × 2	pT1b N0 M0	-	alive, NED

At time of primary diagnosis no metastatic disease was detected in any patient. A definite tumor grading according to accepted grading classifications (Coindre classification) was not applied due to the difficulty of using grading as prognostic factor in ASPS. In one case, however, the tumor was designated a poorly differentiated variant of ASPS (patient 5).

Two patients (patient 1 and 5) had received neoadjuvant therapy (chemotherapy with etoposide, vincristine, adriamycin, ifosfamide and isolated limb perfusion with melphalan and TNF-alpha) prior to surgery because of a large tumor mass adjacent to crucial structures, leaving 70% and 30% of the tumor mass viable in the resection specimen. These two patients were alive with no evidence of disease at follow-up. Incisional biopsy was performed in six cases; fine needle biopsy in one case. Four patients were primarily resected with microscopically positive margins at other institutions and referred to BG-University Hospital Bergmannsheil for curative surgery after histological diagnosis. In all but one case (patient 8), who died of disseminated disease subsequently, free surgical margins were achieved by definite surgery.

All but three patients received adjuvant radiation therapy of the primary tumor site with a dose between 60 and 70Gy. One of these (patient 8) who additionally was not completely resected in the definite operation died of his disease (table 2).

**Table 2:** Summarized patient's and treatment data

Patient	Age/Sex	Untreated tumor growth	Initial procedure	Neodjuvant treatment for primary	Definite procedure	Ajuvant radiation to primary	Local and recurrence treatment	Metastasis and treatment	Status
1	30/F	3 months	incisional biopsy	etoposide, vincristine, adriamycin, ifosfamide	R0 resection	68 Gy	-	-	alive, NED
2	40/M	2 years	fine needle biopsy		R0 resection	60 Gy	-	right lower leg: R0 resection + radiation 60 Gy lung: R2 resection of 21 metastases brain: radiation 2 × 30Gy	DOD
3	21/M	3 years	incisional biopsy		R0 resection	60 Gy	+/adriamycin, ifosfamide	lung: adriamycin, ifosfamide brain: radiation 30 Gy liver: -	DOD
4	26/F	70 years	incisional biopsy		R0 resection	no	-	-	alive, NED
5	30/M	20 years	incisional biopsy	ILP: Melphalan + TNF-alpha	R0 resection	60 Gy	-	-	alive, NED
6	19/F	3 years	R1 resection		R0 resection	no	-	-	alive, NED
7	30/M	2 years	incisional biopsy		R0 resection	65 Gy	-	-	alive, NED
8	48/M	n/a	R1 resection		R1 resection	no	+/R0 resection	lung: epirubicin, ifosfamide brain: R1 resection liver: 5-FU, cisplatin	DOD
9	24/M	6 months	R1 resection		R0 resection	66 Gy	-	no	alive, NED
10	49/M	6 months	R1 resection		R0 resection	66 Gy	-	no	alive, NED
11	34/F	1 month	incisional biopsy		R0 resection	70,4 Gy	-	no	alive, NED

(F: female, M: male, R0-resection: complete resection with free margins, R1-resection: resection with microscopically positive margins, R2-resection: tumor masses remaining in situ, ILP: isolated limb perfusion, NED: no evidence of disease, DOD: died of disease)

**Figure 1:**
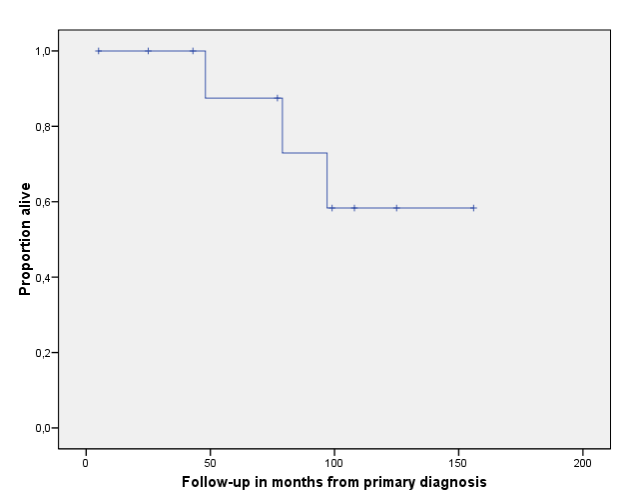
Overall survival after primary diagnosis of ASPS. The tick marks indicate the last follow-up.

Three patients (patients 2, 3, 8) developed metastases in the lung and the brain (n = 3), the liver (n = 2) and the soft tissue (n = 1). Two of those (3 and 8) developed subsequent recurrent disease 29 and 11 months after surgery, which was treated with chemotherapy (patient 3) in one case and lower leg amputation (patient 8) in the other case. Two patients were operated on for their metastases. One patient had the soft tissue metastases (R0) and 21 pulmonary metastases (R2) resected (patient 2); the other one (patient 3) was operated on for his brain metastasis (R1). All intracranial metastases were also treated with adjuvant radiation (30Gy), as well as the one soft tissue metastasis (60Gy). In addition, all patients with metastatic disease received several chemotherapeutics, but unfortunately they all died from disseminated disease after 48, 79, and 97 months. The progression free interval in these patients was 7, 9, and 12 months, respectively (table 3). All other patients were alive with no evidence of disease. The 2 year survival was calculated at 88%, the 5 year survival was calculated 58% (figure 1).

**Table 3:** Time elapsed: Time is calculated from primary diagnosis.

Patient	Time to metastasis (months)	Time to local recurrence (months)	Progression free survival (months)	Follow-up (months)	Time to death (months)
1	-	-	156	156	n/a
**2**	**9**	**-**	**9**	**48**	**48**
**3**	**12**	**29**	**12**	**79**	**79**
4	-	-	43	43	n/a
5	-	-	99	99	n/a
6	-	-	77	77	n/a
7	-	-	125	125	n/a
**8**	**7**	**11**	**7**	**97**	**97**
9	-	-	108	108	n/a
10	-	-	25	25	n/a
11	-	-	5	5	n/a

### Histopathology

**Figure 2:**
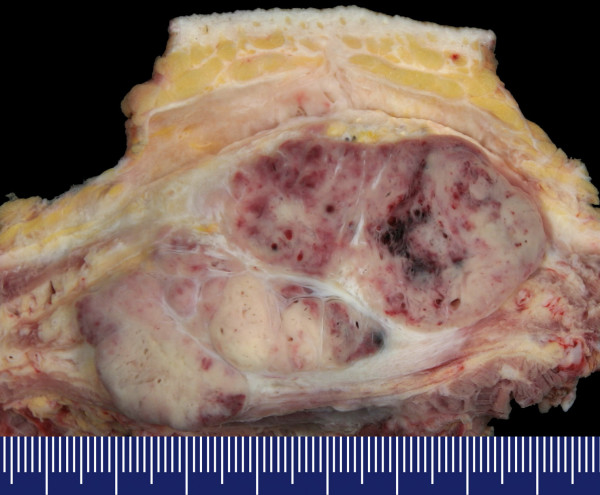
Macroscopic appearance of an alveolar soft part sarcoma, showing a quite solid tumor mass located within the soft tissues. Necrosis is not a striking macroscopic appearance of this sarcoma.

**Figure 3:**
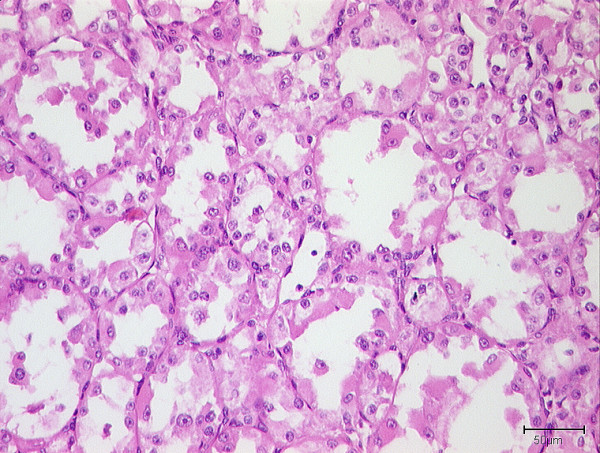
Histologic appearance of an alveolar soft part sarcoma: tumor growth characterized by a central loss of cohesion in cell lobules, depicting a pseudoalveolar archictecture. Round tumor cell lobules, delineated by fibrovascular septa, containing round to polygonally shaped tumor cells with eosinophilic cytoplasm, vesicular nuclei and prominent nucleoli.

**Figure 4:**
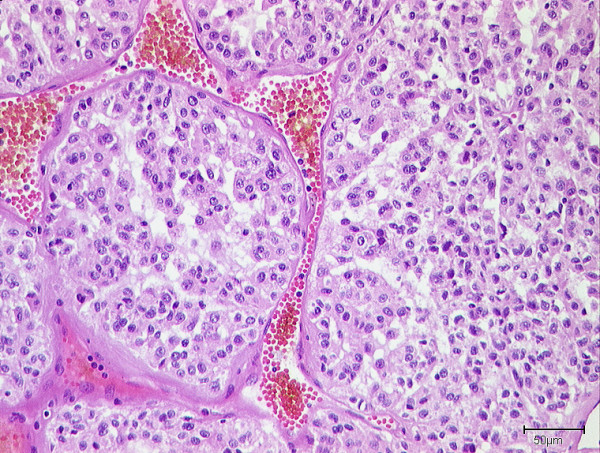
Poorly differentiated case of alveolar soft part sarcoma with overall lobular structure of tumor cell arrangement.

The firm, well vascularized tumors (figure 2) depicted a characteristic alveolar (or pseudo-alveolar) growth pattern (figure 3); the tumor cells being epitheliod and polygonal with eosinophilic cytoplasm, vesicular nuclei and prominent nucleoli. Rhomboid crystalline inclusions could be detected cytoplasmatically. A vascular invasion as a typical finding in alveolar soft part sarcoma was evident in 5 of 11 tumors (figure 4).

In general, most of the tumors showed no or only faint coagulation (tumor) necrosis. Mitotic activity was low (up to 3 mitoses in 10 high power fields), except one case (patient 7) which was characterized by 16 mitoses in 10 high power fields, including atypical ones.

Two patients (patient 1, 5) had been treated systemically before tumor resection; these tumors showed regression ranging from 30–40% vital tumor tissue (patient 5: regression grade IV according to Salzer-Kuntschik) to more than 70% vital tumor (patient 1: regression grade V according to Salzer-Kuntschik).

Crystalline inclusions could be detected in 5 of 11 alveolar soft part sarcomas. Using immunohistochemistry, variable immunohistochemical reactions could be observed with reactivity for S-100 in 2 of 5 examined tumors, focal reactivity for desmin in 4 of 6 tumors, reactivity for actin in 1 of 7 tumors, and weak reactivity for NSE in 1 examined ASPS specimen. No reactivity could be obtained in any tumor for cytokeratins, HMB 45, myogenin, CD 31, CD 34, factor VIII, and synaptophysin.

## Discussion

### Histopathology

The tumor harbors a specific chromosomal translocation at der(17)t(X;17)(p11;q25), often with a loss of the chromosomal region 17q25 (2,5). This translocation results in a fusion of TFE-3-gene (coding for a transcription factor) on chromosome Xp11 and the ASPL (RCC17)-gene of chromosome 17q25. The resulting ASPL-TFE3-oncoprotein causes activation of aberrant transcription [3]. A strong positive immunoreaction against TFE3 (nuclear staining) is characteristic for alveolar soft part sarcoma [1]. Other chromosomal abnormalities like trisomy 7, monosomy 8 and monosomy 18 have also been described [6].

As differential diagnosis especially, metastasis of renal cell carcinoma has to be considered: this possibility can be excluded by history and further clinical and radiological examination, whereby metastasic renal cell carcinoma usually is positive for cytokeratin and vimentine in immunohistochemistry. Other differential diagnoses for the histopathologist include paraganglioma, adrenal cortical carcinoma, hepatocellular carcinoma, alveolar rhabdomyosarcoma, malignant melanoma and granular cell tumor. Paraganglioma may show alveolar structures as well, but, in contrast, is positive by immunohistochemistry for chromogranin and synaptophysin (neuroendocrine markers) and lacks crystalline cytoplasmatic inclusions. Metastases of adrenal cortical carcinoma and hepatocellular carcinoma may be excluded by means of immunohistochemistry (melan-A-cross reactivity in adrenal cortical carcinoma, HepPar in hepatocellular carcinoma) [1]. Alveolar rhabdomyosarcoma exhibits a skeletal muscle differentiation which can be proven by immunohistochemical stains for skeletal-muscle specific markers (myogenin, Myo D1). Malignant melanomas (especially as metastasis) have a growth pattern reminiscent of alveolar structures, but are positive for melanocytic markers like S-100 and HMB 45. A granular cell tumor does not include crystalline bundles and, in contrast to ASPS, is positive for S-100 protein.

Due to the heterogeneity of the immunohistochemical features and the small number of patients, we could not detect a true correlation of histopathological and clinical behavior in our series.

As in other studies with 0.7% of the 1597 soft tissue sarcomas, ASPS represented a very small subgroup. In contrast to previous reports [1,4,5,10], all of our patients were free of lymphatic or detectable hematogenic metastasis at the time of diagnosis and were treated in curative intent. In accordance with the literature, the lesions had been growing to large tumor masses in most of our patients without causing specific symptoms for an extended time and were mostly located in the deep tissues of the thigh [3-5]. The fact that all tumors were located within or close to muscle may support the thesis that the origin of ASPS is myogenic [11,12], although, due to recent genetic findings, a myogenic line of differentiation seems unlikely [7]. Interestingly and contradictory to other reports [1,3,13] the size of the primary at time of diagnosis does not seem to affect the outcome because all three patients who died from their disease had primaries below the average tumor size in our series.

In this study, the age related gender ratio with a male preponderance in older patients -previously noted by Portera [5] and Ordonez [14]) – was also observed. What is more, all patients with an unfavorable outcome were male.

Complete resection of the primary, as well as the recurrent tumor manifestation, seems to be essential for local control [3-5], but cannot prevent distant metastasis in case of early spreading as seen in patient 2 and 3. Recurrent disease – that can be prevented by sufficient primary treatment in about 90% of the cases [1,3,5] – itself seems to be a negative prognostic factor, probably indicating an aggressive tumor biology because all patients with recurrent disease also had distant metastases. Interestingly the metastases occurred before the local recurrence. In these cases, the preferred sequence of manifestation was lung, brain and liver. No isolated brain metastases were observed, whereas there are case reports describing those [15-17] and the large series of 102 patients by Lieberman et. al. mentioned four such cases [4]. As previously reported, lung and brain are the most common sites of metastases [1,16,18] which is why X-ray or CT scans of the lung should be included in the follow-up examinations for ASPS [19]. Considering metastases to the brain almost exclusively occur synchronous or subsequent to pulmonary metastasis, the value of routine intracranial imaging is doubtful and we recommend it only in cases with neurologic symptoms. For the same reason, the same approach is recommended for abdominal MRI or CT scans for liver metastases (with the exception of ultrasound) that are performed at routine follow-ups. Due to the limited number of patients with ASPS, prospective studies would have to span decades to gather a significant collective of patients; therefore, it is not possible to comment meaningfully on a possible benefit of neoadjuvant or adjuvant therapy. In the absence of other data, however, it seems to be justified to recommend postoperative radiation to potentially increase local control rates as in other soft tissue sarcomas. The theory that radiation as single therapy is beneficial for metastatic disease of the brain must be questioned since it seems to be able to slow down growth at best, but failed to induce significant tumor shrinkage in our patients. In accordance with previous medical studies that described limited effects of radiation and chemotherapy on metastatic disease, patients who received such treatment for metastatic disease did not respond in our series[1,3-5,20]. The only patient who received neoadjuvant chemotherapy for the primary also did not respond to it, what was documented by more than 70% of the tumor remaining viable in the resection specimen. Only one patient who was treated with ILP with TNF-alpha and melphalan responded to that treatment, with a necrosis of 70% of the tumor.

The long term outcome of patients with localized ASPS with a 5-year disease free survival rate of 71% [5], 67% [21], and 60% [4], respectively, and a 5-year actuarial survival rate of 88% [5] is considered relatively favorable. From the time of diagnosis of metastatic disease, only about 10% of the patients survive longer than 5 years [4,5]. The clinical courses observed on our series agree with these findings. Although the progression free interval from primary diagnosis to the development of local recurrence or metastasis in three patients was short (7, 9, 12 months), patients lived with metastatic disease but had an acceptable quality of life for a considerable time (39,54,90 months). This prolonged survival with metastatic disease has previously been observed by other authors as well [4,5]. If this long survival time is due to the multimodal therapy or in spite of it cannot be determined thus far.

## Conclusion

In case of acceptable patients' condition and justifiable morbidity, our approach to recurrent disease or resectable solitary metastases continues to be surgical excision followed by radiation with the aim to improve overall quality of life. Chemotherapy is only recommended in selected cases with disseminated disease. Isolated limb perfusion may represent an additional therapeutic option in order to prepare better resection conditions and improve local control in extremity ASPS.

## Competing interests

The authors declare that they have no competing interests.

## Authors' contributions

AD conceptualized the study, gathered the data and wrote the manuscript. CK performed the histopathological evaluation and interpretation of the data. JH analyzed and interpreted the data. OG acquired and weighed the data. DT drafted and revised the manuscript. LS reviewed the literature and analyzed the data. HS conceptualized and supervised the process. He gave final approval for publication. ML initiated the study and drafted the manuscript.

All authors read and approved the final manuscript.
